# Reactive Oxygen Species Imaging in U937 Cells

**DOI:** 10.3389/fphys.2020.552569

**Published:** 2020-10-15

**Authors:** Ankush Prasad, Michaela Sedlářová, Anastasiia Balukova, Alina Ovsii, Marek Rác, Michal Křupka, Shigenobu Kasai, Pavel Pospíšil

**Affiliations:** ^1^Department of Biophysics, Centre of the Region Haná for Biotechnological and Agricultural Research, Faculty of Science, Palacký University, Olomouc, Czechia; ^2^Department of Botany, Faculty of Science, Palacký University, Olomouc, Czechia; ^3^Department of Immunology, Faculty of Medicine and Dentistry, Palacký University, Olomouc, Czechia; ^4^Graduate Department of Electronics, Tohoku Institute of Technology, Sendai, Japan

**Keywords:** U937 cells, hydroxyl radical, oxidative stress, monocytes, immune cells, confocal microscopy

## Abstract

The U937 cell culture is a pro-monocytic, human histiocytic lymphoma cell line. These monocytes can differentiate into either macrophages or dendritic cells (antigen-presenting cells) depending on the initiators. The U937 cells activated in the presence of phorbol 12-myristate 13-acetate (PMA) change their morphology into macrophage-like cells creating pseudopodia and adhering generously. Macrophages are known to produce reactive oxygen species (ROS) mostly during phagocytosis of foreign particles, an important non-specific immune response. Recently, we have focused on the role of hydroxyl radical (HO^∙^) and provide evidence on its importance for differentiation in U937 cells. Based on electron paramagnetic resonance (EPR) spectroscopy combined with confocal laser scanning microscopy (CLSM), formation of HO^∙^ was confirmed within the cells undergoing differentiation and/or apoptosis during the PMA treatment. This study aims to increase our knowledge of ROS metabolism in model cell lines used in human research.

## Introduction

Human myeloid leukemia cell line U937 has been isolated already in the year 1976 from the histiocytic lymphoma of a 37-year-old male (Sundstrom and Nilsson, 1976). The U937 cells grown in suspension are characterized by a round shape, short microvilli, and a large beam-shaped nucleus (Pagliara et al., 2005; Tenuzzo et al., 2008). These monocytic cells bear the potential of differentiating into macrophages or dendritic cells; in response to a number of stimuli adopt the morphology and physiological characteristic of macrophages and thus represent a precursor of mononuclear phagocyte system (Kigerl et al., 2009; Italiani and Boraschi, 2014; Nemati et al., 2019).

Maturation and differentiation of monocytes are influenced by many microenvironmental conditions. Previously, it was reported that U937 cells can be induced to differentiate by several chemical substances including phorbol-12-myristate-13-acetate (PMA), dimethyl sulfoxide (DMSO), retinoic acid, Zn^2+^, 12-O-tetradecanoylphorbol-13-acetate (TPA), and low concentration of glutamine among others (Chun et al., 2001; Yamamoto et al., 2009; Zamani et al., 2013; Mendoza-Coronel and Castanon-Arreola, 2016). The mature differentiated U937 cells attain all the features of a macrophage-like cell which include flat irregular shape, presence of pseudopodia, and phagocytic activity (Whyte et al., 2000; Hsiao et al., 2011). Different concentrations of agents and/or environmental conditions could interfere with the normal cells differentiation processes. Thus, the well-established model system of human leukemia cell line U937 is used that allows detailed investigation of differentiation-dependent alterations (Chanput et al., 2014).

Treatment of U937 cells with PMA, which is capable of directing monocytic cells toward the macrophage pathway, is accompanied by growth arrest and a series of morphological and functional changes (Karina Chimal-Ramirez et al., 2016). The PMA-treated monocyte is referred to as “macrophage-like,” meaning that the properties of the transformed cell line are not yet fully understood (Maess et al., 2014; Lund et al., 2016; Tedesco et al., 2018). These macrophages are clinically significant for possible cancer immunotherapy experimentation (Cassetta and Kitamura, 2018). In the current study, the peculiar morphology of mature macrophages has been investigated by confocal microscopy as well as an attempt has been made to localize the associated ROS formation, specifically hydroxyl radical (HO^∙^). It is known that exogenous addition of PMA can activate nicotinamide adenine dinucleotide phosphate (NADPH)-oxidase which can lead to the formation of superoxide anion radical (O_2_^∙⁣–^) in the cell. Dismutation of O_2_^∙⁣–^ leads to the formation of hydrogen peroxide (H_2_O_2_), and subsequently, it can act as a precursor for the generation of more toxic oxygen compounds, such as HO^∙^ (Auchere and Rusnak, 2002; Halliwell and Gutteridge, 2007; Prasad et al., 2015; Prasad et al., 2016; Pospíšil et al., 2019). To our best knowledge, this is the first report on *in vivo* imaging of HO^∙^ during cell differentiation in U937 human cells.

## Materials and Methods

### Cell Line and Culture Conditions

A human pro-monocytic myeloid leukemia cell line U937 was maintained in suspension culture in RPMI-1640 supplemented with L-glutamine (0.3 g/L), 10% (v/v) heat-inactivated fetal bovine serum (FB-1345), and antibiotics [1% (v/v) penicillin–streptomycin] purchased from Biosera (Nuaille, France). The cells were grown at 37°C in 5% CO_2_ in a humidified atmosphere and subcultured every 3rd or 4th day. Dimethyl sulfoxide was used as an organosulfur compound, polar aprotic solvent for PMA (Sigma Aldrich GmbH, Germany), and its final concentration in cell suspension was always maintained below 1%. U937 cells were induced to differentiate by exposing the cells (5 × 10^5^ cells/mL) with 100 ng/mL of PMA for 0, 24, and 48 h (otherwise stated in the figure legend). Final concentration 100 and 200 nM PMA was based on our previous studies (Kikuchi et al., 2016; Prasad et al., 2016).

### Cell Density and Viability

**FIGURE 1:**
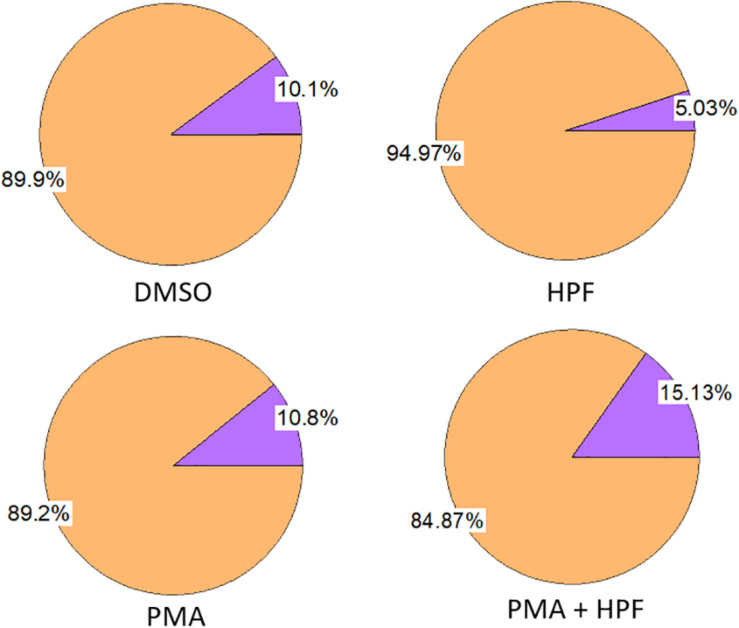
Effect of ≤ 1% dimethyl sulfoxide (DMSO), 1 μM 3’-p-(hydroxyphenyl) fluorescein (HPF), 100 nM phorbol 12-myristate 13-acetate (PMA) and 1 μM HPF with 100 nM PMA on viability of U937 cells as measured following 24 h incubation. The measurement was done with 0.2% trypan blue.

Growth curve of U937 cell culture was monitored during 11 days using TC20 Automated Cell Counter (Bio-Rad Laboratories, Hercules, CA, United States) (Supplementary Data 1) together with cell viability based on trypan blue exclusion. As viable cells do not take up trypan blue (final concentration 0.2%; 2 min incubation; Sigma Aldrich GmbH, Germany) opposite to dead ones, we were able to set the effect of individual chemicals used for solubilization or ROS detection (Figure 1). For subsequent experiments, cells from exponential phase were used in a density of 2.0 × 10^6^ cells/mL.

### Confocal Laser Scanning Microscopy

U937 cells were visualized on slides using Fluorview 1000 confocal unit attached to IX80 microscope (Olympus Czech Group, Prague, Czech Republic). In order to monitor the integrity of cell nuclei and membranes under experimental conditions, co-staining with 2 μM Hoechst 33342 and 15 μM FM4-64 was applied. The excitation of Hoechst 33342 was performed using a 405 nm diode laser and the signal recorded with 430–470 nm bandpass filter, while FM4-64 was excited by a 543 nm He–Ne laser and its emission recorded within 655–755 nm.

Staining of HO^∙^ was based on incubation with 1 μM 3’-p-(hydroxyphenyl) fluorescein (HPF) forming upon reaction with ROS fluorescent product HPFox. Incubation with 1 μM HPF was performed in darkness in the absence or presence of inducer (100 nM PMA) and ranged from 0 to 48 h as described in results. The excitation of HPFox was performed using a 488 nm line of argon laser and the fluorescence was detected by a 505–605 nm filter. Transmitted light detection module with 405 nm diode laser excitation and differential interference contrast (DIC) filters were applied for the imaging of cell morphology. All fluorescent probes were purchased from Thermo Fisher Scientific, Paisley (United Kingdom).

### EPR Spin-Trapping Spectroscopy

Detection of HO^∙^ was performed using an electron paramagnetic resonance spectrometer (MiniScope MS400, Magnettech GmbH, Berlin, Germany) with 50 mM POBN (4-pyridyl-1-oxide-N-tert-butylnitrone) containing 170 mM ethanol (Pou et al., 1994) in PBS saline. The samples were kept in the ultrasonic bath heated up to 37°C for 30 min during the incubation with spin trap (POBN/ethanol) in the absence or presence of inducer (200 nM PMA) in order to increase the penetration of the spin trap through the cell membrane. EPR spectra were recorded under the following conditions: microwave power (10 mW), modulation amplitude (1 G), modulation frequency (100 kHz), sweep width (100 G), scan rate (1.62 G s^–1^).

### Fluorescence Imaging Using Acridine Orange/Propidium Iodide Double Staining

In order to understand the consequence of oxidative stress and to validate our findings, U937 cells were treated with a high concentration of exogenous H_2_O_2_ (5 mM) and incubated in standard conditions for 1 h. Subsequently, the samples were centrifuged at 850 × g for 10 min at 20°C, pellet was rinsed with phosphate buffer saline (PBS) and stained with acridine orange and propidium iodide (AO, 1.5 μM; PI, 1.5 μM). This double staining is a cell viability procedure that causes viable nucleated cells to fluoresce green and non-viable nucleated cells to fluoresce red. Acridine orange permeates viable cells and binds to nucleic acids. PI also binds to nucleic acids, but it is not able to permeate intact cell membranes, therefore is taken up only by non-viable cells and cells with compromised membranes. The maximum excitation/emission wavelengths are 500/526 nm for AO and 533/617 nm for PI. Fluorescence measurements were then performed using Olympus BX51TF (Olympus Corporation, Tokyo, Japan).

## Results and Discussion

### Effect of Solvent and Chemicals on U937 Cell Viability

U937 cells were treated for 24 h in the presence or absence of several chemicals and solvent. Changes in the proportion of viable and dead cells were monitored to understand whether any of the compounds induces considerable cell mortality. The PMA dissolved in a DMSO led to a loss in cell viability by about 10% which is similar as in the case of DMSO alone (Figure 1, left panel). The sample with PMA dissolved in DMSO plus HPF showed a decrease in the proportion of living cells by about 15%, while HPF itself showed a decrease by about 5% (Figure 1, right panel). Application of the chemicals/solvent separately or in combinations maintained the cell viability equal or above 85%, and thus, their impact can be considered as non-significant. It is important to mention that the effect of DMSO on cells and tissues, in general, cannot be ignored as it can have a detrimental effect on the cell morphology and viability, not to mention the preceding finer physiological processes. Preliminary study to set up the final DMSO concentration for a selected cell type is needed at the start of each experiment. In both plant and animal science, the range of 0.5–1% DMSO is being applied to cells, and its final concentration 3% or higher is not advisable (Lee and Park, 2017). In different animal cell lines, it has also been suggested to limit the concentration to a maximum of 1% not only considering its toxicity for the cell lines but also because it can alter a plethora of cell responses (Hollebeeck et al., 2011).

### U937 Cell Differentiation by Phorbol 12-Myristate 13-Acetate

**FIGURE 2:**
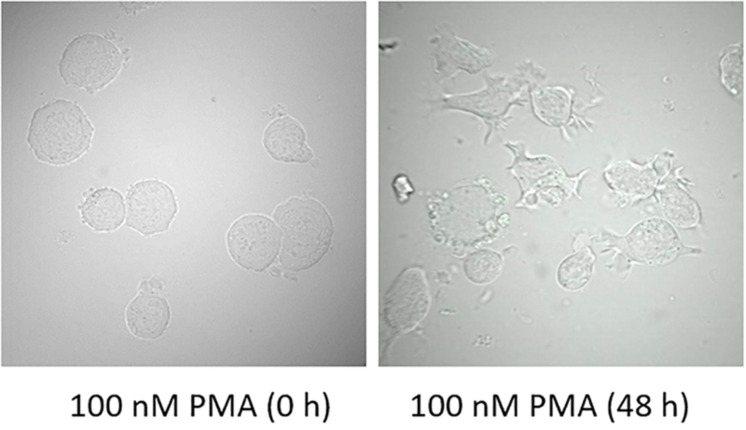
Morphology of U937 cells in the presence of 100 nM PMA at 0 h and 48 h post-incubation at 37°C.

**FIGURE 3:**
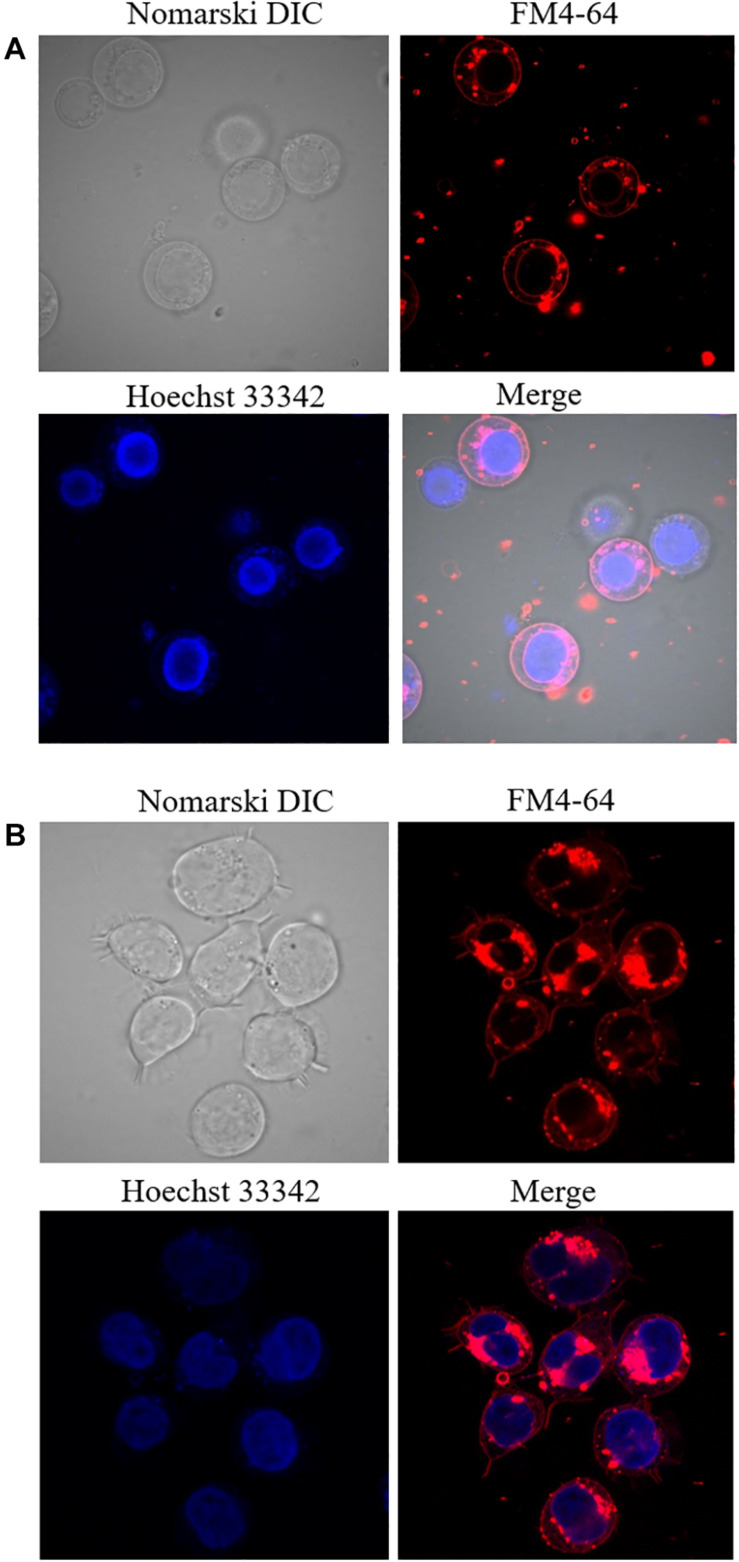
Double staining using Hoechst 33342 and FM4-64 in non-differentiated **(A)** and 48 h differentiated **(B)** U937 cells incubated for 5 min. Images were taken in different channels (Nomarski DIC, FM4-64, Hoechst 33342, and merge of FM4-64 with Hoechst 33342).

U937 cells were treated for two different time intervals and visual changes were monitored at 0 h and 48 h after addition of PMA using a confocal laser scanning microscopy (CLSM). As presented in Figure 2, cell morphology considerably altered following 48 h of incubation with 100 nM PMA. U937 pro-monocytes differentiate into mature monocytes or macrophages upon PMA-treatment, and it has been reported that differentiation is typically observed after 36–48 h; however, it can also depend on the concentration of inducers. During the recent past, phorbol ester and γ-interferon have been studied as two important factors that can stimulate differentiation (Yang et al., 2017). The cell morphology before PMA treatment showed round and translucent structure, while the differentiated cells showed altered morphology, with cells clustering, adhering generously, and forming pseudopodia (Figure 2 and Supplementary Data 2) (Pagliara et al., 2005). Furthermore, in order to validate the cellular integrity of the U937 cells under the experimental condition used, double staining using Hoechst 33342 nucleic acid stain, which is a cell-permeant nuclear counterstain, that emits blue fluorescence following binding to dsDNA and FM4-64, which is a lipophilic styryl compound that emits blue fluorescence when bound to plasma and vesicular membrane, was used (Figure 3). It can be seen that in the case of non-differentiated cells (Figure 3A) and differentiated cell (Figure 3B), cellular integrity was maintained.

### Imaging of Hydroxyl Radical Formation During Cell Differentiation

**FIGURE 4:**
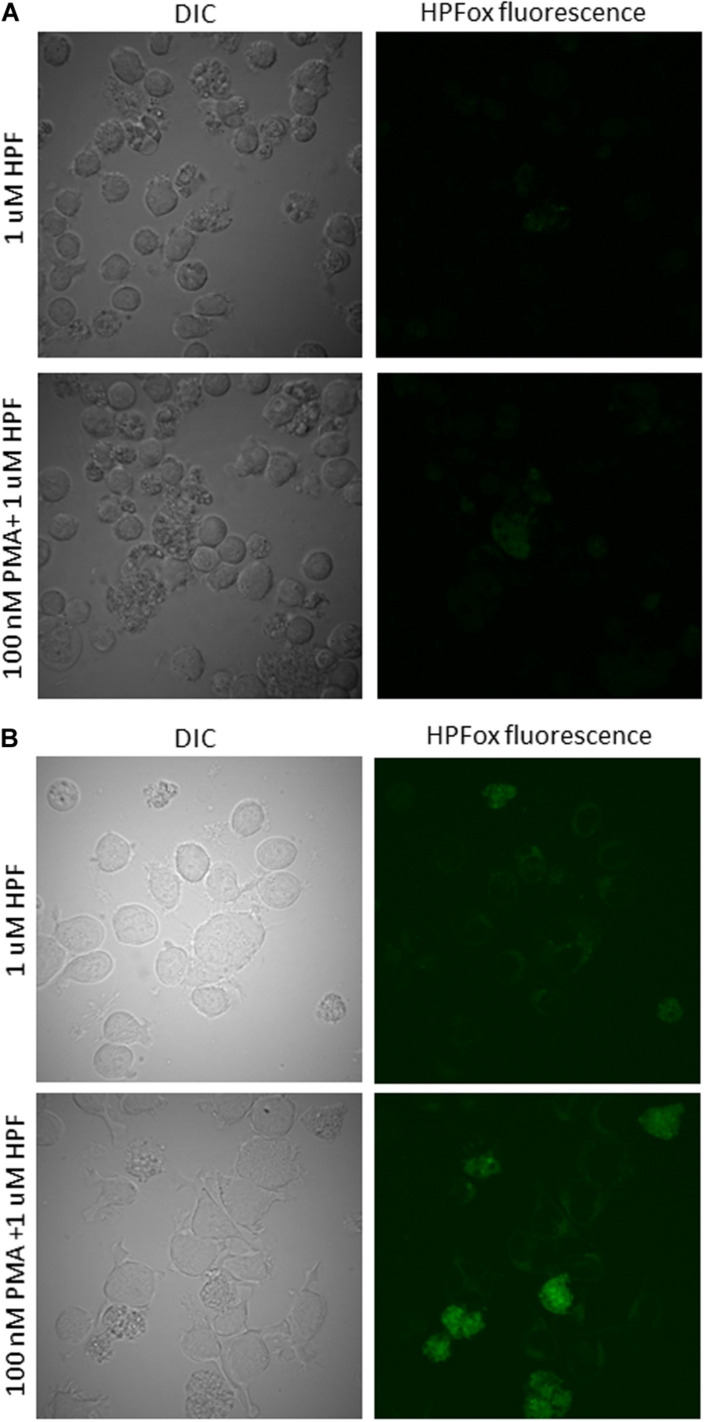
Hydroxyl radical imaging in U937 cells. U937 cells were incubated with 1 μM HPF in the absence (upper panel) and presence (lower panel) of 100 nM PMA dark for 24 h **(A)** and 48 h **(B)**. From left to right are Nomarski DIC channel and HPFox fluorescence.

The confirmation of intracellular ROS generation in human lymphoma U937 cells was done using fluorescent probe HPF for the detection of HO^∙^ after 24 h and 48 h of PMA treatment. It can be seen that following 100 nM of PMA treatment for 24 h in the presence of HPF showed a slight increase in HPFox fluorescence (Figure 4A). The intracellular ROS generation in U937 cells after 48 h was significantly enhanced (Figure 4B and Supplementary Data 3). These results indicate that differentiation and ROS formation are associated phenomenon. Our previous results showed that external oxidative stress can also lead to HO^∙^ formation being tightly linked with the production of singlet oxygen in U937 cells (Rác et al., 2015). Since HPF has been identified to be a probe that can also react with other reactive species including but not limited to peroxynitrite, EPR spectroscopy was needed to validate our results (Dunand et al., 2007; Carlisi et al., 2014; Liu et al., 2018). When other methods are not available, an alternative fluorochrome (for instance, BODIPY) with higher sensitivity to HO^∙^ could be a potential candidate (Lei et al., 2017). However, most fluorochromes suffer certain limitations and have been presented and reviewed in detail and should be used with caution (Rota et al., 1999; Wang et al., 2013; Zeng et al., 2018).

### Detection of Hydroxyl Radical Formation by EPR Spin-Trapping Spectroscopy

**FIGURE 5:**
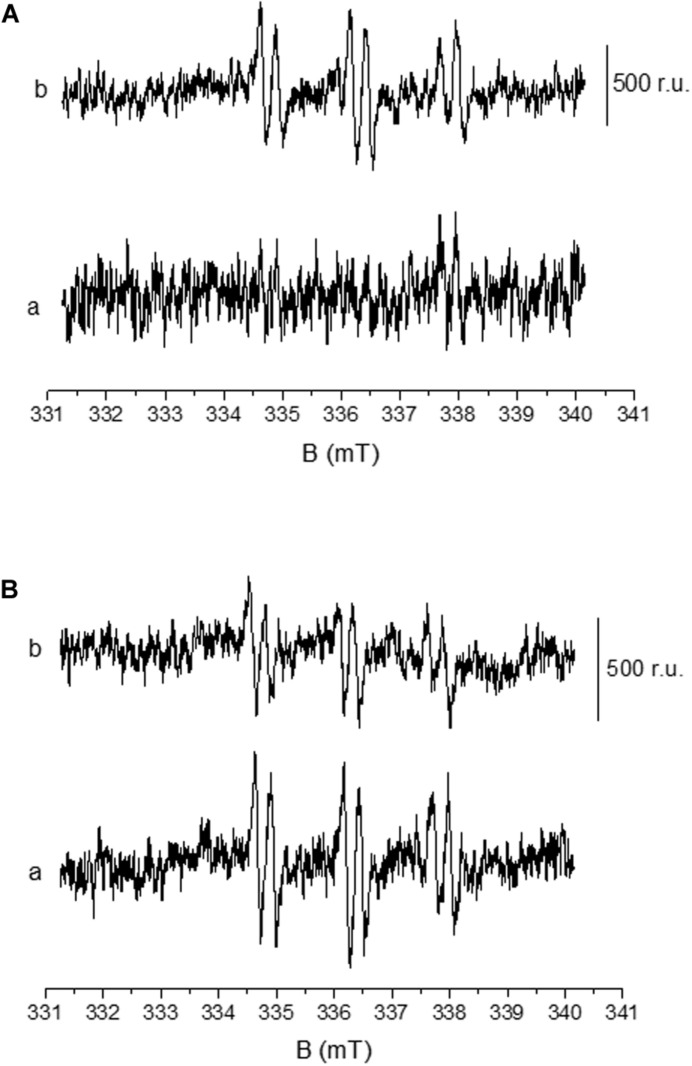
Detection of HO^∙^ using EPR spin-trapping spectroscopy in U937 cells. PMA induced POBN (4-pyridyl-1-oxide-N-tert-butylnitrone)-CH(CH_3_)OH adduct EPR spectra: Differentiated U937 cells with 50 mM POBN in the absence (**A**, trace a) and presence of 200 nM PMA (**A**, trace b). POBN-CH(CH_3_)OH adduct EPR spectra were measured after 30 min of sonication performed in the presence of 200 nM PMA. The lower panel **(B)** shows the EPR spectra as in **A** (trace b) in the absence (trace a) and presence of 400 U/mL superoxide dismutase (SOD).

Formation of HO^∙^ in U937 cells was measured by EPR spectroscopy using POBN/ethanol spin trap system. No or negligible intensity of EPR signal was observed in control U937 cells (Figure 5A, trace a), whereas treatment with 200 nM PMA to differentiated cells for 30 min resulted in the formation of the α-hydroxyethyl radical adduct of POBN [POBN-CH(CH_3_)OH adduct] EPR signals (Figures 5A, trace b, and B, trace a). It can be seen that the addition of superoxide dismutase (SOD) prior to PMA treatment led to significant suppression of EPR signal (∼50%) (Figure 5B, trace b) which is in agreement with the results presented by Zeng et al. (2018). Data simulation was performed and presented as Supplementary Data 4. The current observation validates the formation of HO^∙^ during the progress of differentiation in U937 cells.

### Detection of Cell Damage Using Fluorescence Microscopy

**FIGURE 6:**
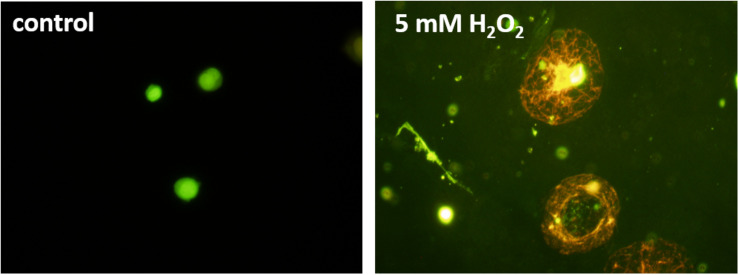
Fluorescence images of U937 cell culture in the absence (**left panel**) and under the effect of 5 mM hydrogen peroxide (H_2_O_2_) added exogenously. Acridine orange/propidium iodide staining was used for staining the cells.

For the detection of cell damage, AO/PI co-staining was utilized as an indicator of membrane integrity. While AO can penetrate the cell membranes of viable cells making them fluorescence green, PI is impermeable to alive cells and stains nucleic acids of damaged or dead cells resulting in red fluorescence (Liu et al., 2015; Rosenberg et al., 2019). Due to the Förster resonance energy transfer, the PI signal absorbs the AO signal in dead cells, ensuring no double-positive results. Figure 6 shows a healthy U937 cell (control) and cells under 5 mM H_2_O_2_. The healthy cells on the left can be seen to provide green fluorescence, while the damaged/dead cells are stained red. It can also be observed that the non-treated cells still hold their shape, while the H_2_O_2_ treated cells have changed morphology and show an increase in the size, most likely due to the damaged cell membranes.

## Conclusion

While more studies are necessary to comprehend the details of the effects under investigation, it nevertheless appears clear that the differentiation of U937 cells to monocytes is accompanied by the formation of ROS. We used confocal laser scanning microscopy to visualize the formation of HO^∙^ and validated the results using EPR spectroscopy. Since PMA can also be involved in producing a cascade of oxidant species by the activity of myeloperoxidase and nitric oxide synthase, the possibility and potential generation of other ROS is possible in addition to HO^∙^ and should be taken into account. The use of confocal imaging opens new opportunities to study the localization and overall understanding of the role of ROS in signal transduction.

## Data Availability Statement

All datasets presented in this study are included in the article/Supplementary Material.

## Author Contributions

AP, SK, and PP contributed to the conceptualization of the study. AP, MS, AB, AO, MR, and MK contributed to the data curation. AP contributed to the formal analysis, validation, and writing of the original draft. AP, MS, SK, and PP contributed to the writing (review and editing). All authors contributed to the article and approved the submitted version.

## Conflict of Interest

The authors declare that the research was conducted in the absence of any commercial or financial relationships that could be construed as a potential conflict of interest.

## Supplementary Material

Supplementary Data 1 Growth curve of U937 cell following seeding at a density of 1.17 × 10^5^ cells/mL.

Supplementary Data 2 Focusing through a non-differentiated **(A)** and differentiated **(B)** U937 cell. The differentiation of the cell was induced using 100 nM PMA for 48 h. The video represents a series of 9 **(A)** and 11 **(B)** optical sections (*x*,*y*) through a sample sequentially acquired at *z* = 1 μm.

Supplementary Data 3 Hydroxyl radical imaging in U937 cells. U937 cells were incubated with 1 μM HPF in the absence (**upper panel**) and presence (**lower panel**) of 100 nM PMA dark for 48 h. From left to right are Nomarski DIC channel and HPFox fluorescence.

Supplementary Data 4 PMA induced POBN (4-pyridyl-1-oxide-N-tert- butylnitrone)-CH(CH_3_)OH adduct EPR spectra as in Figure 5A (trace b) and simulated POBN-CH(CH_3_)OH adduct EPR spectrum obtained using the hyperfine coupling constants *a*^*N*^ = 15.75 G and *a*^*H*^ = 2.40 G.

## Abbreviations

ROS: reactive oxygen species
EPR spectroscopy: electron paramagnetic resonance spectroscopy
CLSM: confocal laser scanning microscopy
PMA: phorbol 12-myristate 13-acetate.
