# Supplementary figures and images for: Reactive Oxygen Species Imaging in U937 Cells
Source: Front Physiol. 2020 Oct 15;11:552569. doi: 10.3389/fphys.2020.552569 (PMC7593787; doi:10.3389/fphys.2020.552569)

Supplementary data 1

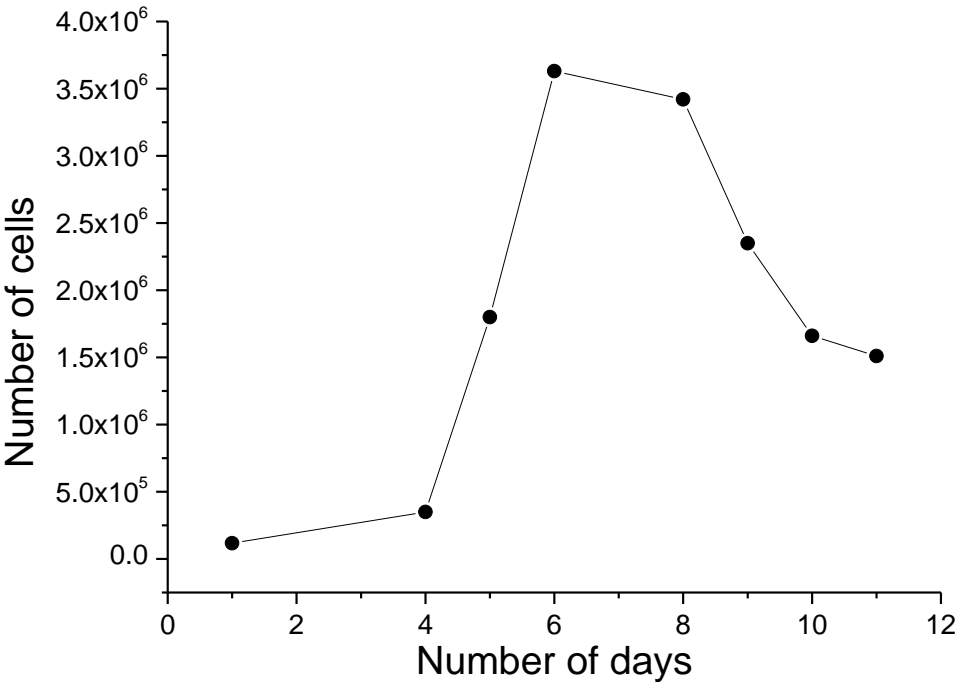

Supplement: Supplementary Data 1 — Growth curve of U937 cell following seeding at a density of 1.17 × 10^5^ cells/mL. [file Data_Sheet_1.PDF]

Supplementary data 3

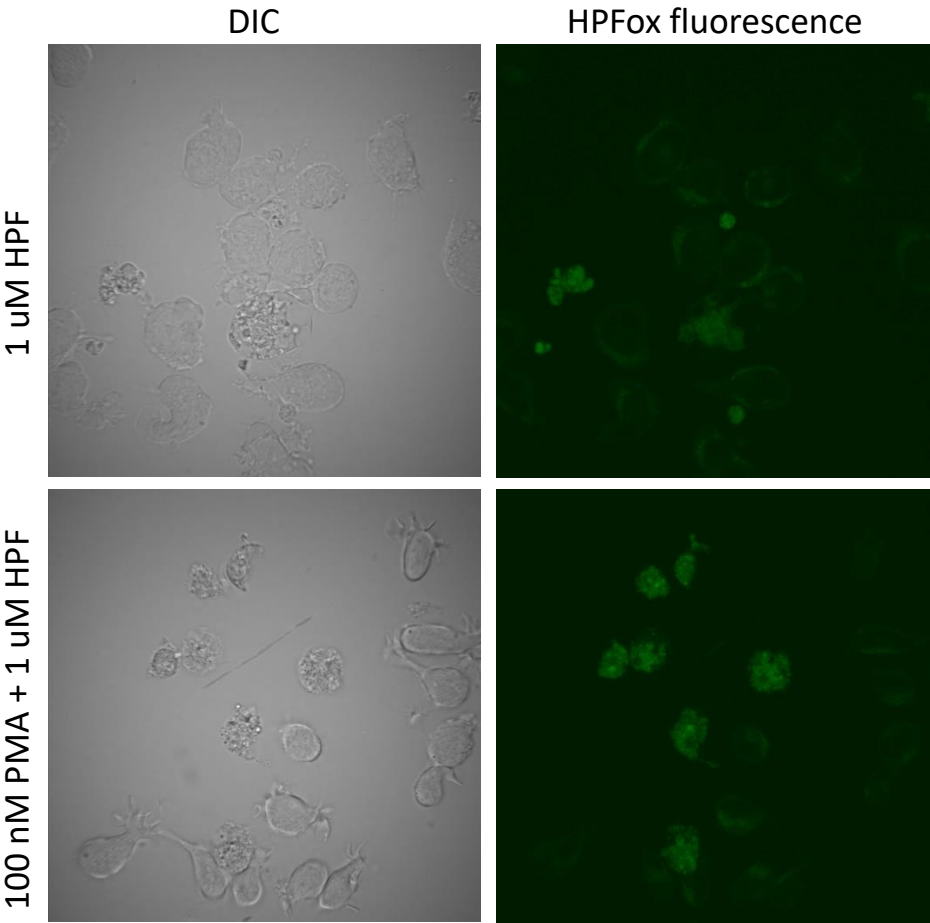

Supplement: Supplementary Data 3 — Hydroxyl radical imaging in U937 cells. U937 cells were incubated with 1 μM HPF in the absence (upper panel) and presence (lower panel) of 100 nM PMA dark for 48 h. From left to right are Nomarski DIC channel and HPFox fluorescence. [file Data_Sheet_3.PDF]

Supplementary data 4

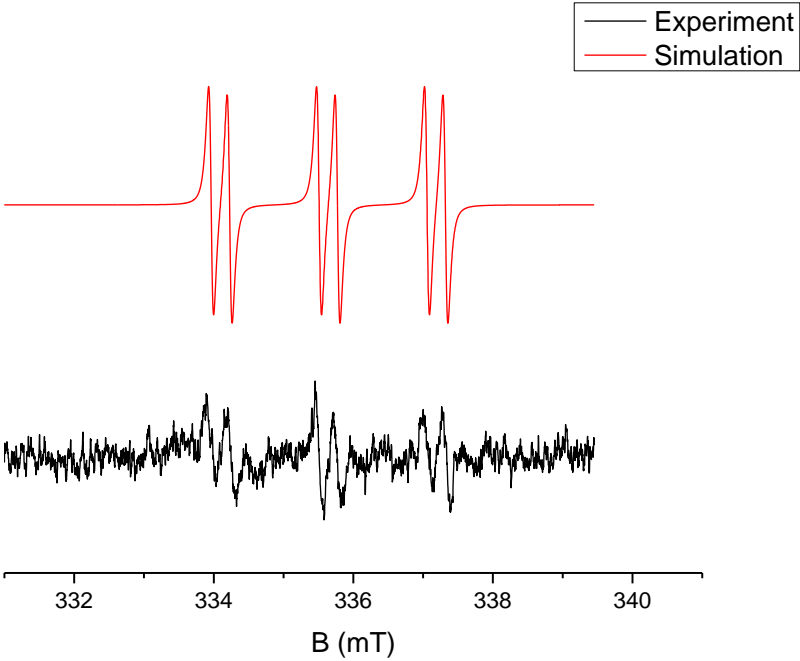

Supplement: Supplementary Data 4 — PMA induced POBN (4-pyridyl-1-oxide-N-tert- butylnitrone)-CH(CH3)OH adduct EPR spectra as in Figure 5A (trace b) and simulated POBN-CH(CH3)OH adduct EPR spectrum obtained using the hyperfine coupling constants aN = 15.75 G and aH = 2.40 G. [file Data_Sheet_4.PDF]
